# Thyroblastoma Dominated by a Primitive Small Round Cell Component: A Diagnostic Pitfall

**DOI:** 10.1007/s12022-026-09916-0

**Published:** 2026-04-15

**Authors:** Atsuko Kasajima, Marco M. E. Vogel, Günter Klöppel, Abbas Agaimy, Costanza Chiapponi

**Affiliations:** 1 Department of Pathology, Klinikum rechts der Isar, TUM School of Medicine and Health, Technical University of Munich, Munich, Germany; 2 Department of Radiation Oncology and Radiation Therapy, Klinikum rechts der Isar, TUM School of Medicine and Health, Technical University of Munich, Munich, Germany; 3 Department of Pathology, Friedrich-Alexander-University Erlangen-Nürnberg, Erlangen, Germany; 4 Department of Surgery, Klinikum rechts der Isar, TUM School of Medicine and Health, Technical University of Munich, Munich, Germany; 5 Bavarian Center for Cancer Research (BZKF), Munich, Germany

**Keywords:** Thyroblastoma, Small blue round cell tumor, Differential diagnosis

## Case History

A 46-year-old woman presented with a long-standing thyroid nodule that had rapidly enlarged in recent months. Evaluation showed a 2.5 cm nodule in the left thyroid lobe and three enlarged ipsilateral cervical lymph nodes up to 3 cm. Intraoperative frozen sections of the thyroid nodule and one lymph node revealed a malignant small round blue cell tumor, prompting left hemithyroidectomy with central and lateral lymphadenectomy.

## What is your Diagnosis?

**Fig. 1:**
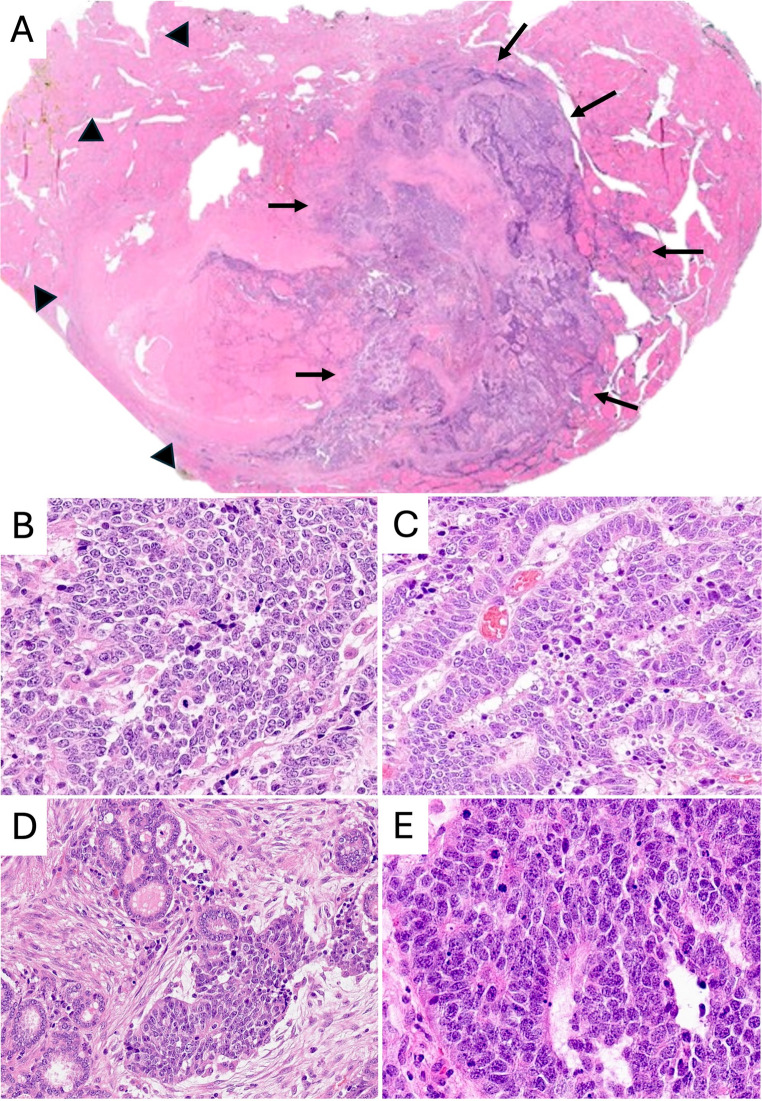
(**A**) A 1.8 cm tumor within a 2.5 cm sclerotic, encapsulated thyroid nodule (arrowheads), with capsular invasion and parenchymal infiltration (arrows). (**B**) Solid growth of small round to oval cells with enlarged nuclei, scant cytoplasm, coarse chromatin, and brisk mitoses. (**C**) Focal tubule-like structures of columnar cells. (**D**) Tumor nests partly embedded in fibrous stroma with focal connection to follicles. (**E**) Lymph node metastases with solid nests and multiple rosettes, lacking follicular or spindle components

The tumor was predominantly located within a 2.5 cm sclerotic thyroid nodule, showing capsular invasion and infiltration into the surrounding thyroid parenchyma (Fig. 1A). Histologically, it consisted of solid sheets of small round to oval cells with enlarged nuclei, scant cytoplasm, and high mitotic activity (Fig. 1B). Focal tubule-like structures were present (Fig. 1C), with partial embedding in fibrous stroma and focal continuity with thyroid follicles (Fig. 1D).

Three lateral cervical lymph nodes showed metastases composed of small round cells with multifocal rosettes (Fig. 1E), abundant necrosis, and high mitotic activity, without stromal or follicular/tubular differentiation.

**Fig. 2:**
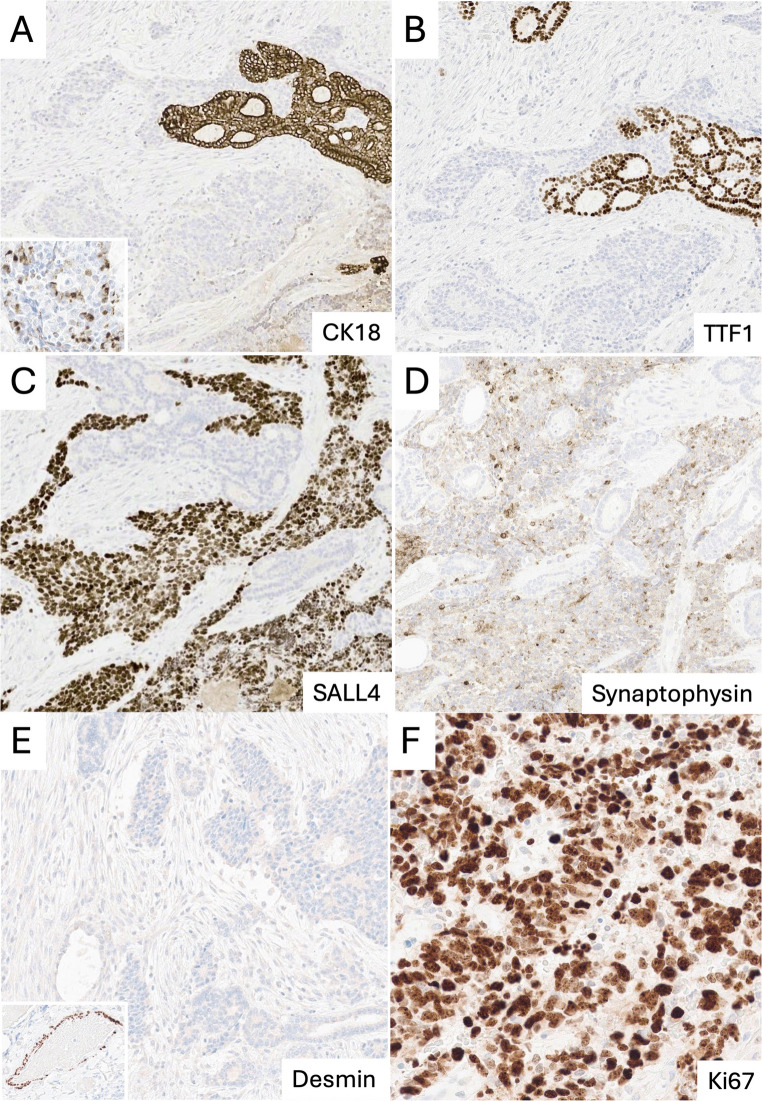
Immunohistochemistry: (**A**) Tumor cells largely CK18-negative with focal perinuclear positivity (inset); follicles strongly positive. (**B**) TTF1 positive in follicles, negative in tumor cells. (**C**) Strong nuclear SALL4 expression. (**D**) Weak, heterogeneous synaptophysin expression. (**E**) No desmin-positive stromal cells (vascular control, inset). (**F**) Very high Ki-67 proliferation

Immunohistochemically, tumor cells were largely cytokeratin-negative with focal CK18 positivity (Fig. 2A), and negative for TTF1, PAX8, and thyroglobulin (positive in adjacent follicles; Fig. 2B). SALL4 was strongly expressed (Fig. 2C), synaptophysin was weak and heterogeneous (Fig. 2D), and chromogranin A and INSM1 were negative.

No stromal expression of desmin (Fig. 2E) or myogenin was detected. Tumor cells were also negative for calcitonin, S100, CD99, SOX10, CD45, CEA, BRAFV600E, NUT, and OCT4. Nuclear SMARCA4 expression was retained, and p53 and retinoblastoma protein (RB1) showed a normal expression pattern. The proliferative index was very high (> 90%, Ki-67; Fig. 2F).

Targeted next-generation sequencing identified two pathogenic DICER1 variants: a splice-site mutation (c.5603 + 2T > C, variant allele frequency 40%) and an RNase IIIb hotspot mutation (c.5127T > A; p.D1709E, 37%) consistent with a typical two-hit mechanism.

## Diagnosis

Thyroblastoma with a prominent blastemal component.

## Comments

Thyroblastoma is a rare, high-grade thyroid neoplasm with a typically triphasic architecture: primitive small cells, a sarcomatous component (often rhabdomyosarcoma-like), and primitive follicular structures [1]. The small-cell component shows strong SALL4 expression, while the sarcomatous component expresses myogenic markers such as desmin or myogenin [2].

In this case, the tumor consisted predominantly of the primitive small-cell component, lacking a sarcomatous or cartilaginous component. Immature follicles were not clearly identifiable, likely due to infiltration of adjacent thyroid tissue. Lymph node metastases contained only the small-cell component, obscuring the typical triphasic pattern.

In this setting, the differential diagnosis includes primary or metastatic neuroendocrine carcinoma of the thyroid. Both may show small round blue cell morphology, rosette-like structures, and high proliferation. However, neuroendocrine carcinomas typically show diffuse expression of markers such as synaptophysin, chromogranin A, and INSM1. In contrast, thyroblastomas usually show absent or only focal, weak neuroendocrine marker expression but strong nuclear SALL4 positivity, reflecting primitive embryonal differentiation. Lack of calcitonin expression further argues against medullary thyroid carcinoma.

Other important differentials include NUT carcinoma, which shows a broad histologic spectrum [3], as well as anaplastic thyroid carcinoma and lymphomas with entrapped follicles. Additional considerations are small round cell tumors such as Ewing sarcoma or related undifferentiated sarcomas, which may show focal cytokeratin or synaptophysin positivity but typically lack chromogranin A and thyroid lineage markers [4].

Thyroblastoma is strongly associated with pathogenic DICER1 alterations, often combining a loss-of-function mutation with an RNase IIIb hotspot mutation, supporting the diagnosis [2, 5].

## Data Availability

No datasets were generated or analysed during the current study.

## Declarations

### Concent for Publication

All authors consent to publication.
